# Supplementary material for: Sensing force and charge at the nanoscale with a single-molecule tether
Source: Nanoscale. 2021 Jul 5;13(29):12687–96. doi: 10.1039/d1nr01970h (PMC8319944; doi:10.1039/d1nr01970h)
Supplement: NR-013-D1NR01970H-s001 [file NR-013-D1NR01970H-s001.pdf]

Electronic Supplementary Material (ESI) for Nanoscale.
This journal is © The Royal Society of Chemistry 2021

Supplementary information

Sensing force and charge at the nanoscale with a single-molecule tether

Xuanhui Meng,[1] *Philipp Kukura*[1,*] *and Sanli Faez*[2,*]

[1]Physical and Theoretical Chemistry Laboratory, University of Oxford, South Parks Road OX1 3QZ Oxford, U.K.

[2]Nanophotonics, Debye Institute for Nanomaterials Research, Utrecht University, NL

ASSESSING LOCALISATION PRECISION

To assess the localisation precision (LP) of our dark-field setup, we followed the approach by Webb et al.[1] The experimental LP is characterised with sparse 20 nm gold nanoparticles immobilised on the glass surface. Every particle is localised and tracked in order to obtain its position, which is then plotted as a function of time. To remove the contributions by overall sample drift and vibrations, the distance between each pair of particles is calculated and the localisation precision is determined from the standard deviation of the fluctuations in the distance between two particles divided by a factor of $\sqrt{2}$, since each measurement involves the localisation of two beads. By quantifying the localisation precision at different illumination intensities (or exposure times), the localisation decays as $\sqrt{N}$ as expected[1] (Fig. S1a).

A similar assessment is performed to guide us to set an optimal exposure time when carrying out force measurement where the averaged displacement is of interest. A molecular tether is specifically anchored on the coverslip surface. When the exposure time is too short, the measurement error is dominated by nanoparticle diffusive motion. If we continue to increase the shutter speed, at some point, motion blurring comes into play and begins to deteriorate the accuracy. As shown in Fig. S1b, after characterising the measured LP at various exposure times with a fixed incident light intensity the camera exposure time was set to 2 ms to ensure the highest tracking precision.

DOUBLE-STRANDED DNA TEHTERS

The double stranded DNA construct of 160 bp is prepared by annealing the two complementary, single DNA strands. The dried single-stranded oligonucleotides functionalised at their 5'-ends with biotin are reconstituted in 10 mM HEPES buffer and mixed. The mixtures are heated quickly to 90°C followed by a cooling rate of 1°C/min down to 25°C in a polymerase chain reaction (PCR) machine, resulting in a 5 μM dsDNA solution. The sequence of the single-stranded oligonucleotides with 50% GC content is listed below:

5′ biotin- CTG GAT GTG TGA TGT AGC GCA CTG ATT CTA TAT GGA TAC CTA GAG TTG CCT ATA CCT CGA CCA GAC CAA GGG TGA GTG GTT GCA GTG TGA TCC TAG AGG GGC CAG AGG AGA TCG TAC ATT ATT GGG TGC CTT CCT GGG TAA GTG AGA TAC CTG TGA GAA C -3′

5′ biotin- GTT CTC ACA GGT ATC TCA CTT ACC CAG GAA GGC ACC CAA TAA TGT ACG ATC TCC TCT GGC CCC TCT AGG ATC ACA CTG CAA CCA CTC ACC CTT GGT CTG GTC GAG GTA TAG GCA ACT CTA GGT ATC CAT ATA GAA TCA GTG CGC TAC ATC ACA CAT CCA G -3′

ZETA-POTENTIAL MEASUREMENTS

The zeta-potential of streptavidin-coated 20 nm gold nanoparticles were measured with a Zetasizer (Malvern Nano Z). Solutions of 20 nm AuNPs were prepared in working buffer (150 mM KCl, 10 mM HEPES) at pH 5.5 and 7.6, and the concentration is ~10 pM for both samples. At 25°C, the zeta-potentials in pH 5.5 and pH7.6 WB were found to be -8.9 mV and -30.8 mV, respectively, which support the observed displacements shown in Figure 6b. At pH 5.5, each tethered particle only carries a few charges and is nearly electrically neutral,[2] while the particle displacements are considerably larger at pH 7.6, increasing from 1.51 nm to 5.84 nm, corresponding to a 32.8 mV change of the zeta potential (Particle No.2 in Figure 6b).

CURRENT MEASUREMENT

Because of the rate-limiting effects at the electrodes,[3] the actual EF is more related to the current that passes through the channel than the applied potential. To estimate the actual EF correctly we monitored the current using a 100-ohm resistor. The actual EF is determined from the measured current density and solution conductivity with $E = J/\sigma$. A drawing of all the circuit shown in Figure S2a specifies where probes are placed.

- CH1 is the measured voltage (x10 probe) directly from the piezo driver which is the same port that applies the potential to the flow chamber

- CH2 is the pulsed signal for driving blue laser as reference beam

- CH3 is the measured voltage (x10 probe) across the chamber and resistor

- CH4 is the measured voltage across the resistor.

CORRECTION OF CAMERA FRAME RATE

The actual frame rate is set by custom-written LabVIEW software. To verify the actual frame rate, we pre-set the camera frame rate to 250 frames per second (FPS) and recorded a sequence of images with illumination laser flashing at 2 Hz. The intensity variation of one camera pixel, is plotted in Fig. S3a. The Fast Fourier transform analysis in Fig. S3b shows a frequency of 2.3 Hz of the light source. Therefore, we could calculate the actual camera frame rate as 250 FPS * 1 Hz / 2.3 Hz = 108.7 FPS. By confirming the source of discrepancy, we extract the actual frame rate for all measurements based on the reference pulsed laser (445 nm laser flashing at 1 Hz generated via Digilent Analog Discovery 2) in each movie individually. After correction, as shown in Fig. 3c the spectrum peaks at 0.47 Hz, which is in good agreement with the applied potential modulation.

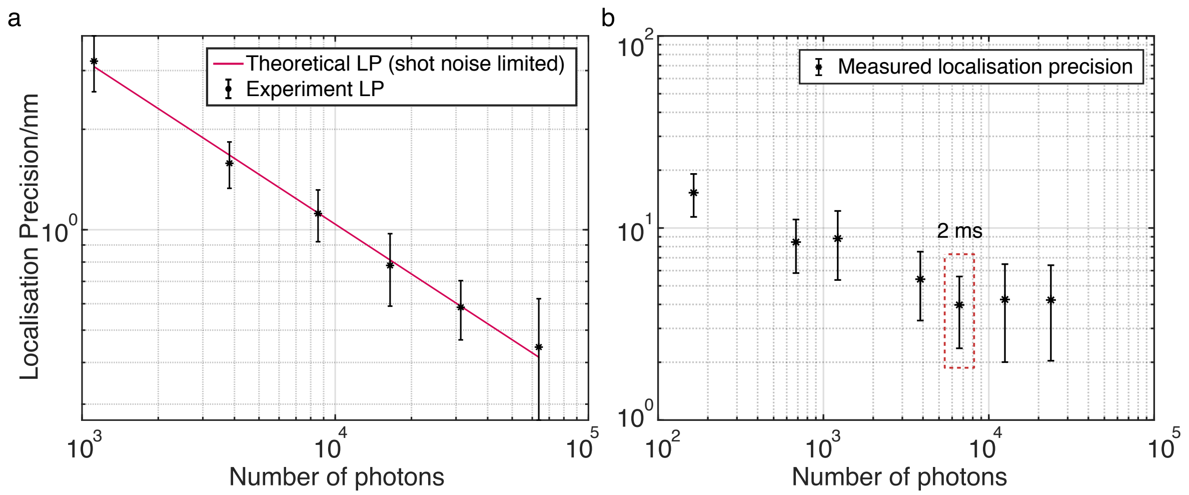

Figure S1. Characterisation of localisation error. (a) Calibrated experimental localisation precision and theoretical limits dependence on camera exposure time for 2.7 kW/cm^2 incident illumination density. (b) Given a fixed incident light intensity, localisation error is re-characterised to determine the optimal camera exposure time to ensure the highest tracking precision by averaging out the nanoparticle diffusive motion.

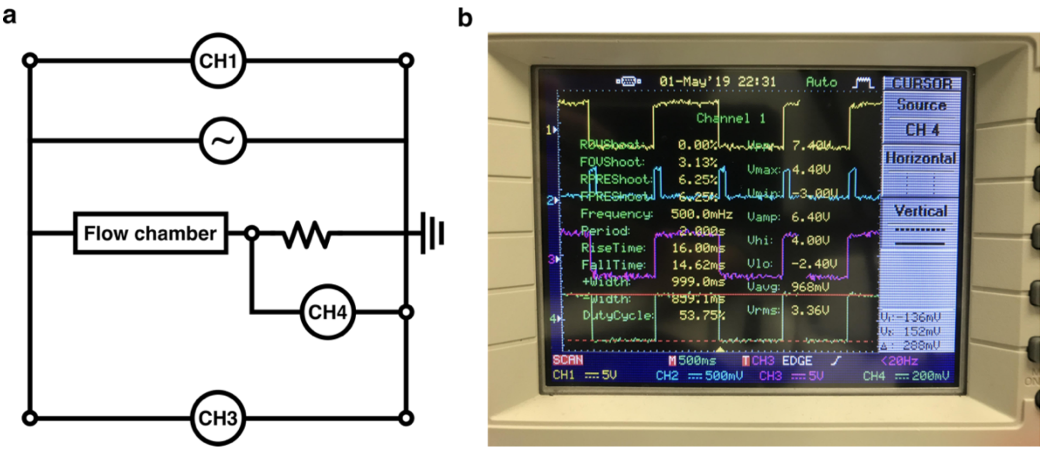

Figure S2. Monitoring current across the flow chamber. (a) Schematic showing where probes are places. (b) A photo of oscilloscope screen presents voltages of all detection channels. A constant number of the voltage (CH4) across resistor supports that the actuall EF remains stable over measurements.

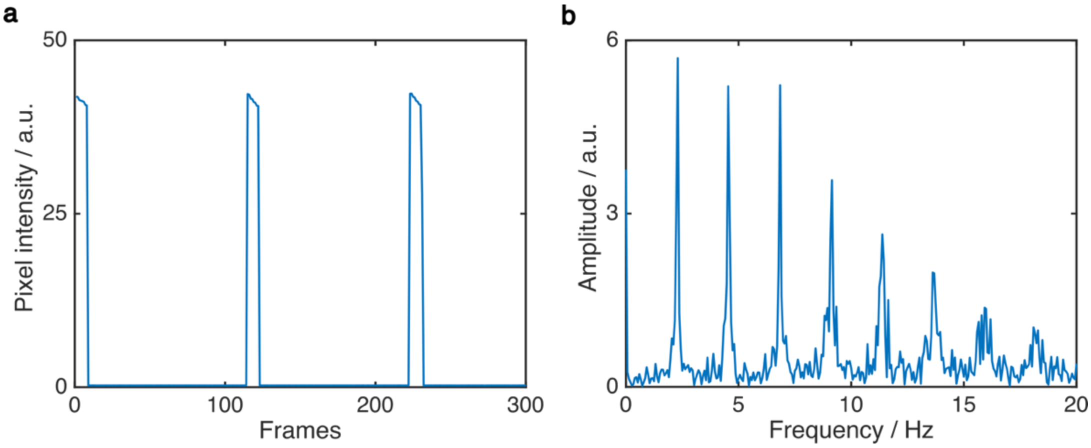

Figure S3. Verification of camera frame rate. (a) Reconstructed signal from recorded data of illumination beam flashing at 1 Hz with 10% duty cycle. Figure is plotted using one-pixel intensity variation vs. time. (b) The FFT analysis of illumination beam reveals a frequency of 2.3 Hz.

REFERENCE

(1) Thompson, R. E.; Larson, D. R.; Webb, W. W. Precise Nanometer Localization Analysis for Individual Fluorescent Probes. *Biophys. J.* **2002**, *82*, 2775–2783.

(2) Diamandis, E. P.; Christopoulos, T. K. The Biotin-(Strept)Avidin System: Principles and Applications in Biotechnology. *Clin. Chem.* **1991**, *37*, 625–636.

(3) Hall, S. B.; Khudaish, E. A.; Hart, A. L. Electrochemical Oxidation of Hydrogen Peroxide at Platinum Electrodes. Part II: Effect of Potential. *Electrochim. Acta* **1998**, *43*, 2015–2024.
